# The Distribution and Possible Roles of Small Cardioactive Peptide in the Nudibranch *Melibe leonina*

**DOI:** 10.1093/iob/obaa016

**Published:** 2020-05-29

**Authors:** W H Watson, A Nash, C Lee, M D Patz, J M Newcomb

**Affiliations:** 1 Department of Biological Sciences, University of New Hampshire, Durham, NH 03824, USA; 2 Department of Biology and Health Science, New England College, Henniker, NH 03242, USA

## Abstract

The neuropeptide small cardioactive peptide (SCP) plays an integrative role in exciting various motor programs involved in feeding and locomotion in a number of gastropod species. In this study, immunohistochemistry, using monoclonal antibodies against SCP_B_, was used to localize SCP_B_-like-immunoreactive neurons in the central nervous system, and map their connections to various tissues, in the nudibranch, *Melibe leonina*. Approximately 28–36 SCP_B_-like-immunoreactive neurons were identified in the *M. leonina* brain, as well as one large neuron in each of the buccal ganglia. The neuropil of the pedal ganglia contained the most SCP_B_-like-immunoreactive varicosities, although only a small portion of these were due to SCP_B_-like-immunoreactive neurons in the same ganglion. This suggests that much of the SCP_B_-like immunoreactivity in the neuropil of the pedal ganglia was from neurons in other ganglia that projected through the pedal–pedal connectives or the connectives from the cerebral and pleural ganglia. We also observed extensive SCP_B_ innervation along the length of the esophagus. Therefore, we investigated the impact of SCP_B_ on locomotion in intact animals, as well as peristaltic contractions of the isolated esophagus. Injection of intact animals with SCP_B_ at night led to a significant increase in crawling and swimming, compared to control animals injected with saline. Furthermore, perfusion of isolated brains with SCP_B_ initiated expression of the swim motor program. Application of SCP_B_ to the isolated quiescent esophagus initiated rhythmic peristaltic contractions, and this occurred in preparations both with and without the buccal ganglia being attached. All these data, taken together, suggest that SCP_B_ could be released at night to arouse animals and enhance the expression of both feeding and swimming motor programs in *M. leonina*.

## Synopsis

El neuropéptido pequeño péptido cardioactivo (SCP) juega un rol integrativo en inducir varios programas motores involucrados en la alimentación y locomoción de varias especies de gasterópodos. En este estudio se usó inmunohistoquímica con anticuerpos contra SCP_B_ para localizar y describir la conectividad de neuronas con inmunoreacción semejante al SCP_B_ en el sistema nervioso central del nudibranquio *Melibe leonina*. Identificamos entre 28 y 36 neuronas en el cerebro de *M. leonina* con inmunoreacción semejantes al SCP_B_, además de una neurona grande en cada ganglio bucal. El neuropilo del ganglio pedal contiene la mayor cantidad de varicosidades neuronales con inmunoreacción semejante al SCP_B_ aunque sólo una fracción pequeña de éstas provenían de neuronas en el mismo ganglio. Esto sugiere que gran parte de la inmunoreacción semejante al SCP_B_ en el neuropilo del ganglio pedal tienen su origen en neuronas en otros ganglios que enviaron sus proyecciones a través de los conectivos entre los ganglios pedales, o provenientes de los ganglios cerebral o pleural. Observamos además extensa inervación con immunoreacción semejante al SCP_B_ a lo largo del esófago. Por lo tanto investigamos el impacto de SCP_B_ en la locomoción de animales intactos, así también como las contracciones peristálticas en preparaciones de esófagos aislados. Inyecciones nocturnas de SCP_B_ en animales intactos resultó en in incremento significante en rastreo y nado comparado con animales inyectados con soluciones salinas. Más aún, perfusión de cerebros aislados con SCP_B_ inició programas de programas motores de nado. La aplicación de SCP_B_ a preparaciones aisladas de esófagos inactivos inició contracciones peristálticas rítmicas, lo cual ocurrió igualmente en preparaciones con conexiones con el ganglio bucal presentes o ausentes. Tomados en conjunto, nuestros datos sugieren que SCP_B_ podría ser liberado de forma nocturna para despertar los animales y aumentar la expresión de programas motores de alimentación y locomoción en *M. leonina*.

Neuropeptidet, small cardioactive peptide (SCP), spiller en vigtig roller i forbindelse med kontrollen af motorikken under fødeoptag og bevægelse hos flere snegle. Dette studie brugte immunohistokemi med monoklonale antistoffer mod SCP_B_ til at lokalisere SCP_B_ lignende immunoreaktive neuroner i centralnervesystemet, samt til at kortlægge deres forbindelser til forskelligt væv i nøgensneglen, *Melibe leonina.* Omkring 28-36 SCP_B_ lignende immunoreaktive neuroner blev fundet i *M. leonina* hjerne sammen med en stor neuron i hvert buccal ganglie. Neuropilet i fod gangliet indeholdt flest SCP_B_ lignende immunoreaktive varikositier, men det kun var en mindre del af dem, som skyldes SCP_B_ lignende immunoreaktive neuroner i det samme ganglie. Dette antyder at megen af SCP_B_ lignende immunoreaktiviteten i neuropilet i fod gangliet var fra neuroner i andre ganglier, som har udløbere gennem fod-fod nervebanerne eller i nervebaner fra cerebrale og pleurale ganglier. Vi observerede også udbredt SCP_B_ innervering langs svælget. Derfor undersøgte vi både effekten af SCP_B_ på bevægelsen af intakte dyr og på peristaltikken af isolerede svælg. Injektion af SCP_B_ i intakte dyr om natten resulterede i dyr, som svømmede og kravlede signifikant mere end kontrol dyr, som fik injektion med saltvand. Perfusion med SCP_B_ i isolerede hjerner medførte desuden igangsættelse af det motoriske program for svømning. Når SCP_B_ blev tilsat til ubevægelige svælg startede rytmiske peristaltiske bevægelse, og dette skete i præparater med og uden buccal gangliet vedhæftet. Når al data sammenholdes indikerer det, at SCP_B_ frigøres om natten for at aktivere dyrene og øge aktiviteten af de motoriske programmer bag både fødeoptag og svømning i *M. leonina*.

## Introduction

Neuropeptides constitute a large class of diverse signaling molecules with a wide range of functions in both the endocrine and nervous systems (Burbach 2011). In order to characterize the role of these molecules as neuromodulators, several species of gastropod mollusks have served as effective species with which to study how neuromodulators can act on individual neurons and neural networks (Harris-Warrick and Marder 1991; Jing et al. 2009). The primary advantage of these species is that they have very large, identifiable neurons, which are amenable to neurophysiological studies.

A well-studied group of neuropeptides in gastropods is the small cardioactive peptides (SCP_A_ and SCP_B_; Lloyd 1982) that were first sequenced from *Aplysia californica* (Morris et al. 1982; Lloyd et al. 1987). SCP has excitatory effects on the heart in *A. californica*, *Archidoris montereyensis*, *Helix aspersa*, and *Limax maximus* (Lloyd 1980; Lloyd et al. 1985; Welsford and Prior 1991; Lesser and Greenberg 1993; Wiens and Brownell 1995). However, although it has cardio-excitatory actions, we are not aware of any evidence to suggest that this class of neuropeptides is involved in the normal regulation of cardiac activity (Lloyd 1982).

In addition to having cardio-excitatory actions, SCP_B_ has been shown to play an important role in modulating gastropod gut motility (Lloyd et al. 1988a), feeding motor programs (Watson and Willows 1992), and aspects of feeding responsiveness (Murphy et al. 1985; Prior and Watson 1988). Additionally, immunohistochemical studies have demonstrated the presence of SCP_A_ and SCP_B_ neurons in the buccal ganglia, which are involved in the control of feeding and swallowing behaviors (Lloyd et al. 1984a; Watson and Willows 1992).

The nudibranch *Melibe leonina* is a promising animal for investigation of modulatory neuropeptides, such as SCP_B_, because the neural mechanisms underlying several behaviors have been studied, including for feeding, swallowing, and swimming (Trimarchi and Watson 1992; Watson and Willows 1992; Watson et al. 2001, 2002; Thompson and Watson 2005; Sakurai et al. 2014). *Melibe leonina* are more active at night (Newcomb et al. 2004) and this pattern of activity is due to an endogenous circadian clock (Newcomb et al. 2014; Cook et al. 2018; Duback et al. 2018), which may capitalize on neuromodulatory systems to adjust the likelihood of certain behaviors being expressed. Furthermore, while there has not been a thorough investigation of SCP_B_ localization or function in *M. leonina*, previous studies do indicate that this neuropeptide is present in at least a few select neurons (Watson and Willows 1992; Lillvis et al. 2012).

The overall goal of this study was to determine the possible roles of SCP_B_ in *M. leonina*. Specifically, this study had three major objectives: (1) to map the distribution of SCP_B_ in the central nervous system (CNS) of *M. leonina* using immunohistochemistry; (2) to determine if SCP_B_ influences locomotion; and (3) to determine if SCP_B_ influences peristaltic contractions of the esophagus.

## Materials and methods

### Animal care

Some experiments were carried out at Friday Harbor Laboratories (FHL), while others were performed at the University of New Hampshire (UNH). For experiments at FHL, *M. leonina* were collected by the authors from eelgrass beds just offshore from Shaw Island. Animals were housed in outdoor tanks that were perfused with flow through seawater and at ambient conditions. *Melibe leonina* fed on zooplankton present in the seawater. For experiments at UNH, *M. leonina* were collected from kelp beds in Monterey Bay, CA, by Monterey Abalone Company, and shipped overnight to UNH. Animals were housed in tanks at 10–12°C and exposed to a light regime of 12 h of light, followed by 12 h of darkness. *Melibe leonina* were fed *Artemia* nauplii two to three times a week. Animals were chilled prior to any experiment requiring an incision, to reduce injury-induced firing of sensory receptors.

### Immunohistochemistry

*Melibe leonina* brains and attached buccal ganglia were removed, along with the esophagus, and pinned in a Sylgard-lined glass dish in seawater. The immunohistochemistry protocol followed procedures previously described (Watson and Willows 1992; Newcomb et al. 2006). First, the tissues were fixed overnight in 4% paraformaldehyde in phosphate buffered saline (PBS) at 4**°**C. Fixed preparations were then washed three times in PBS, transferred into 1.5 mL Eppendorf tubes, and incubated in 4% Triton-X in PBS for 1 h. During this step, and all incubations, the Eppendorf tubes were placed in a rack on a slow-moving shaker table and kept at 12**°**C. The tissues were then blocked in 0.5% Triton-X in PBS, containing 6% goat serum (PBSTG), for 1–2 h, and then incubated with primary antibodies diluted 1:20 in PBSTG for ∼2 days. Dr. Dennis Willows provided the monoclonal SCP_B_ antibodies, raised in mice (see Masinovsky et al. (1988) and Watson and Willows (1992) for previous studies with these antibodies, including preadsorption controls).

Following the 2-day incubation with primary antibodies, the brains and associated tissues were washed three times in 0.5% Triton-X in PBS and blocked again in PBSTG for 2–6 h. Goat anti-mouse secondary antibodies conjugated to Alexa 488 or Alexa 555 fluorophores were then added to the solution at a 1:100 dilution and the tissues were incubated for another 24–48 h. Finally, the preparations were washed in PBS for 1–3 h before being mounted on glass slides using Fluoromount aqueous mounting medium.

Slides were viewed and photographed with either a Zeiss Axioplan epifluorescence microscope or a Zeiss LSM 510 Meta laser scanning confocal microscope. Images obtained on the confocal microscope used an argon multiline 488 nm excitation filter for Alexa 488 and helium-neon 543 nm filter for Alexa 555. A consensus map of SCP_B_-like-immunoreactivity in the brain and buccal ganglia of the *M. leonina* was created by averaging the number of neuronal cell bodies viewed in the 11 preparations with the highest signal-to-background ratio.

### Retrograde tracing with Neurobiotin

Immunohistochemical processing of the *M. leonina* CNS with SCP_B_ antibodies revealed intense staining of the neuropil in the pedal ganglia that seemed to arise from axons in the pedal–pedal connective (PPC). To test this hypothesis, *M. leonina* brains were removed and the PPC was cut close to one of the pedal ganglia, leaving the remainder of the PPC to backfill. The brains were then placed into individual petroleum jelly wells in a Petri dish. The severed end of the PPC was carefully placed over the edge of the well and into a second, adjacent well. Seawater was used to submerge the brain in one well and several drops of distilled water were placed into the well containing the end of the PPC nerve. The end of the nerve was then cut again and left in the distilled water for ∼30 s. The distilled water was then replaced with a 4% solution of Neurobiotin (NB) in 1 M KCl. Parafilm was stretched over the top of the Petri dishes and a cover was placed over the Parafilm to reduce evaporation of the solutions. The preparations were then incubated at 4**°**C for 12 h.

The brains were then removed from the wells, pinned in a glass dish, washed several times with PBS, and then processed according to the procedure described above for SCP_B_ immunohistochemistry. However, Steptavidin-Alexa Fluor 594 conjugate, at a 1:50 dilution, was added during the secondary antibody step, in addition to the goat anti-mouse secondary antibodies conjugated to Alexa Fluor 488. This allowed us to visualize both SCP_B_ neurons and neurons with axons in the PPC to determine if they overlapped, indicating that SCP_B_ neurons projected through the PPC to the pedal ganglion on the opposite side of the brain.

### Effects of SCP_B_ on locomotion

*Melibe leonina* were housed at 16**°**C on a 12:12 light–dark cycle (daylight from 6 am to 6 pm). Individual animals were placed in one of three containers that were situated within a larger tank of seawater. The water in the larger holding tank was aerated with an air pump and filtered with activated carbon to reduce ammonia levels. The three containers had small holes to enable water exchange with the larger tank. This arrangement reduced ripples and bubbles in the individual containers, making it easier to see the animals and analyze the time lapse videos.

A low-light video camera was suspended over the three containers and its output was digitized and then recorded at 1 frame/s using Gawker. The videos were then analyzed using Ethovision XT to determine the distance traveled by each of the *M. leonina* throughout the study. These data were then compiled in Excel and used to calculate the distance each animal moved every 30 min.

To determine if SCP_B_ had an impact on locomotion, a total of nine animals were used for this experiment, with six injected with either saline or SCP_B_ in the daytime, and three animals injected with either saline or SCP_B_ at night. In the night experiment, the three animals were placed in the chambers described above and allowed to adjust to the tank for one day. We then recorded 24 h of continuous time lapse digital video data and during this time period, they were each injected at 6:30 pm with 0.5 mL of 0.5 M NaCl, as a control, using a 22-gauge needle and 1-mL syringe. Then, after recovering for a day, we repeated the process, but this time they were injected at 6:30 pm with 10^−5^ M SCP_B_, dissolved in 0.5 M NaCl. In this experiment, and the others described below, preliminary experiments were done with SCP_B_ at concentrations ranging from 10^−4^ M to 10^−7^ M and we ultimately used 10^−5^ M SCP_B_ because it was the lowest concentration that yielded reliable effects.

The daytime experiments were conducted in the same manner, except that animals were injected at 7:00 am. The videos that were obtained, were analyzed as described above to yield, for each animal, distance moved per 30 min, during the 3 h after they were injected with either saline or SCP_B_.

### Effects of SCP_B_ on the swim motor pattern

*Melibe leonina* brains were removed and pinned out in a dish that was continuously perfused with cooled (12°C) seawater. Expression of the swim motor program was monitored with suction electrodes attached to the smaller of the two branches of the Pd4 nerve that originates in the pedal ganglia (Newcomb and Watson 2001). The signals from these nerves were amplified with an A-M Systems Model 1700 AC amplifier and then digitized and displayed with an ADI Powerlab 2/26, and an Apple iMac computer running LabChart software. Swimming was manifested as rhythmic bursts with an interburst interval of ∼2–3 s, and bursts in the left Pd4 and right Pd4 out of phase with each other (because they swim by bending their bodies with alternating left–right lateral flexions). Small (100 µL) aliquots of 10^−5^ M SCP_B_ dissolved in seawater were added to the recording chamber and, because of the continuous perfusion, washed off soon after addition. The recording chamber held ∼20 mL, so the SCP_B_ was diluted ∼200-fold.

### Effects of SCP_B_ on the esophagus

To examine the effects of SCP_B_ on the esophagus, it was removed from the animal and pinned in the same recording dish used for the extracellular recordings described above. In some cases, the buccal ganglia were left on the esophagus so that it would contract spontaneously, while in other preparations, the buccal ganglia were removed so that the esophagus rarely contracted on its own. A small pin, which was on a string connected to a force transducer (Grass Instruments, FTO3), was used to pierce the lateral portion of the esophagus to monitor contractions. The output of the force transducer was amplified and digitized with an ADI Powerlab 2/26, and then displayed and recorded with LabChart software running on an Apple iMac computer. After obtaining a stable baseline recording for at least 10 min, 100–500 µL of 10^−5^ M SCP_B_, dissolved in 0.5 M NaCl, was added to the recording chamber. Because the preparations were being continuously perfused with seawater, the SCP_B_ was rapidly diluted, and hence effects did not last long.

## Results

### Immunohistochemistry and retrograde tracing with NB

**Fig. 1:**
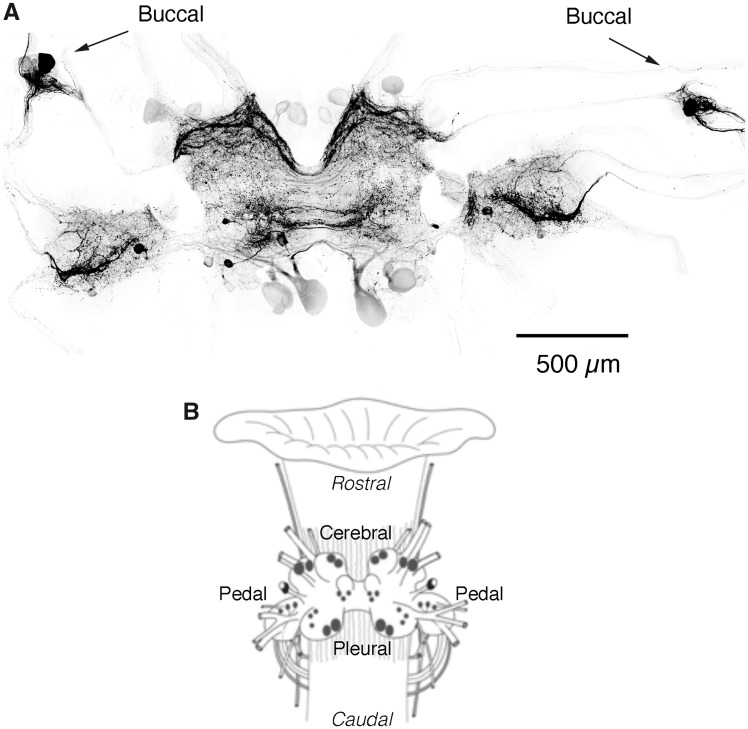
SCP_B_-like-immunoreactive neurons and processes in the CNS of *M. leonina*. (**A)** A confocal montage of SCP_B_-like immunoreactivity (black), comprised of smaller high resolution images. Note the single large cell body in each of the buccal ganglia (arrows), as well as the extensive SCP_B_-likeimmunoreactive projections throughout the brain. (**B)** Diagram of a *M. leonina* brain attached to the esophagus, illustrating the location of SCP_B_ neurons in the *M. leonina* CNS. Typically, SCP_B_ antibodies labeled 13–17 cell bodies in the paired cerebropleural ganglia, 2-4 SCP_B_ cell bodies in each of the pedal ganglia, and 1 SCP_B_ cell body in each of the buccal ganglia.

**Fig. 2:**
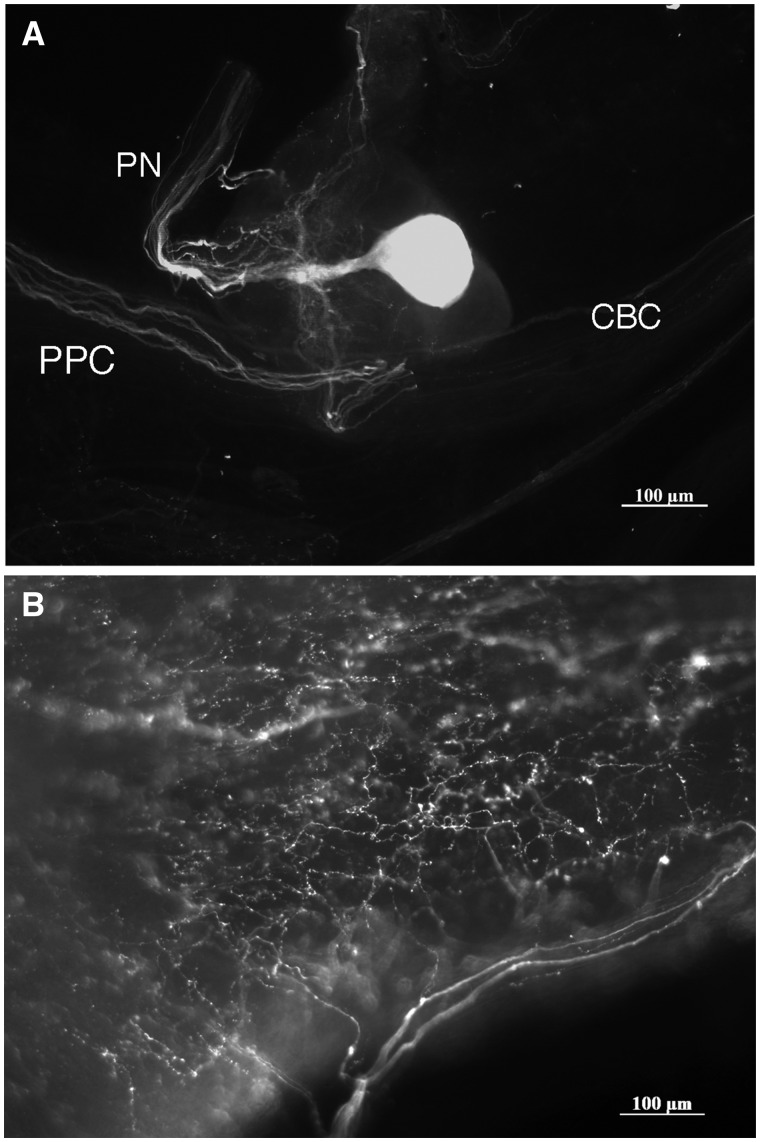
An SCP_B_-like-immunoreactive neuron in one of the buccal ganglia (**A**) and its processes on the esophagus (**B**). The large SCP_B_-containing neuron sends most of its projections out of the posterior nerve and to the other buccal ganglion via the buccal–buccal connective. CBC = cerebral-buccal connective; PN = posterior nerve; PPC = pedal–pedal connective (not part of the buccal ganglion or its nerves).

There were 20.6 ± 8.3 (mean ± standard deviation; *n* = 11) SCP_B_-like-immunoreactive neurons present in the *M. leonina* brain and one large SCP_B_ neuron in each of the buccal ganglia (Figs. 1 and 2). Four large neurons (50–100 µm in diameter) in the anterior region of each cerebral ganglion and two large neurons in the posterior region of each pleural ganglion exhibited faint SCP_B_ labeling, but with defined projections. Three to six smaller but more strongly labeled SCP_B_-like-immunoreactive neurons were present in the center region of each cerebropleural ganglion. Another one to three smaller neurons in each pedal ganglion also exhibited SCP_B_-like immunoreactivity. The large SCP_B_-like-immunoreactive neurons in the buccal ganglia project to various areas along the esophagus (Fig. 2), as well as to a pair of small ganglia that reside at the junction between the esophagus and stomach (Trimarchi and Watson 1992). SCP_B_-like-immunoreactive fibers were found in cerebral nerves 1-4, pedal nerves 1-5, and pleural nerves 1-2 (Table 1; nerves numbered according to Hurst (1968)).

**Table 1:** Nerves containing SCP_B_-like-immunoreactive axons, and the tissues to which they connect (based on Hurst 1968)

Nerve	Brain origin	Target tissue
C1	Cerebral	Mouth
C2	Cerebral	Anterior of oral hood
C3	Cerebral	Middle of oral hood
C4	Cerebral	Rhinophore
Pd1	Pedal	Foot
Pd2	Pedal	Anterior foot
Pd3	Pedal	Posterior foot
Pd4	Pedal	Lateral body wall and foot
Pd5	Pedal	Foot
P1	Pleural	Digestive and reproductive
P2	Pleural	Digestive tract

**Fig. 3:**
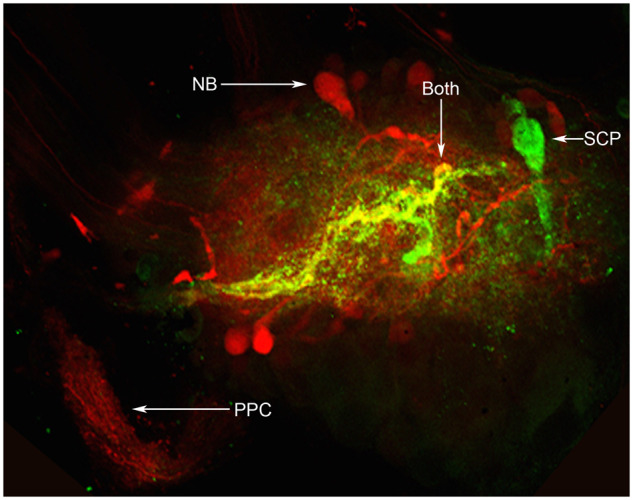
Combination of NB (red) backfill of the PPC and SCP_B_ immunohistochemistry (green) in the pedal ganglion. There were numerous neurons that gave rise to axons in the PPC but did not exhibit SCP_B_-like immunoreactivity, and these were backfilled with NB and labeled red. There were also several SCP_B_-positive neurons that projected to the neuropil of the pedal ganglion (center) but not out of the PPC and thus labeled green. However, there were 1-2 SCP_B_-like-immunoreactive neurons (just one is visible in this focal plane of this preparation) that projected axons to the contralateral pedal ganglion via the PPC and therefore labeled yellow.

In addition to numerous SCP_B_-labeled cell bodies, SCP_B_-like-immunoreactive processes were found throughout the neuropil of the brain and buccal ganglia and the antibody labeling was most intense in the pedal ganglia. It appeared as if many of the processes and varicosities in the pedal ganglia originated from axons in the PPCs. When these were backfilled with NB and then brains were co-stained with SCP_B_ antibodies, just 1-2 neurons contained both SCP_B_ and NB and projected to the opposite pedal ganglia (*n* = 8; Fig. 3).

### Effects of SCP_B_ on locomotion

**Fig. 4:**
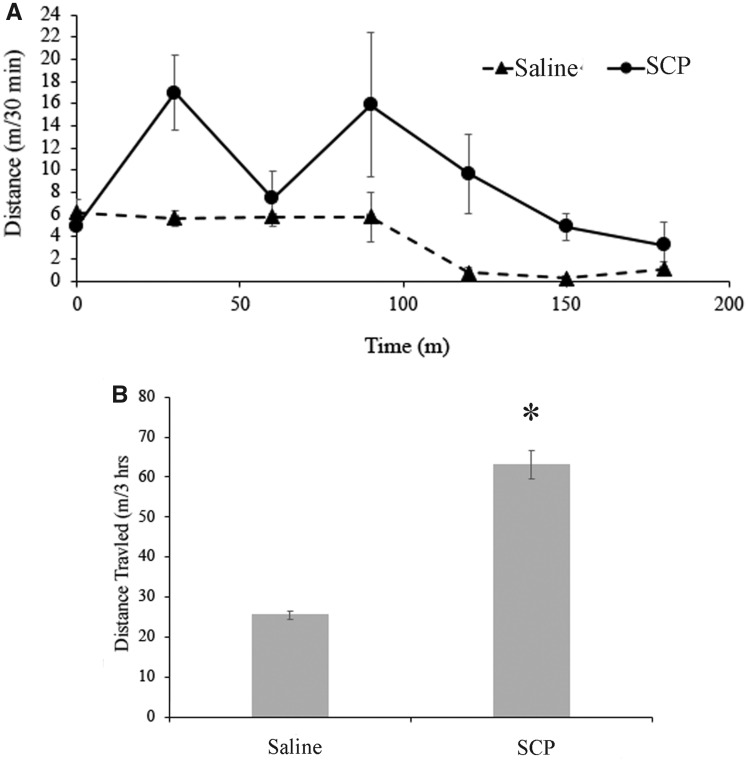
Influence of SCP_B_ on locomotion in *M. leonina* at night. (**A)** Average activity levels (meters crawled/30 min) were elevated for several hours after injection with SCP_B_, compared to saline injections in these same animals (*n* = 3). (**B**) Animals injected with SCP_B_ moved significantly more in the 3 h after they were injected (63.1 ± 3.6 m) than the same animals after they were injected with a saline control (25.5 ± 1.0 m) (*P* = 0.0047).

**Fig. 5:**
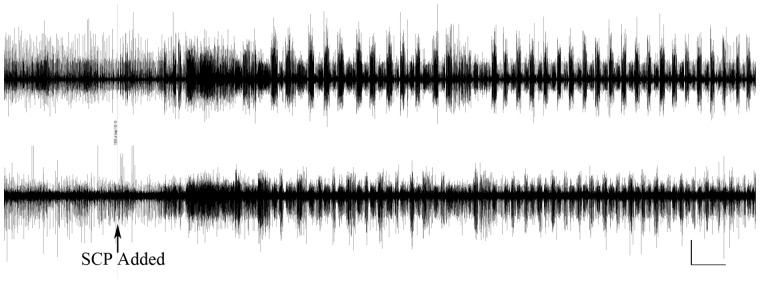
The impact of SCP_B_ on the swim motor program expressed by the isolated *M. leonina* brain. Extracellular recordings were obtained from both the left and right pedal nerve 4. The addition of SCP_B_ (100 ul of 10^−5^ M added to a dish containing 20 mL of seawater), elicited expression of the swim motor program, characterized by rhythmic bursting of the motorneurons that would normally cause the *M. leonina* to bend laterally side-to-side. Scale bars: 10 s, 20 uV.

All animals that were injected with saline (*n* = 9), or SCP_B_ (the same animals, *n* = 9), responded with a brief period of swimming. However, when they were injected with SCP_B_ at the beginning of the night (Fig. 4; *n* = 3), they were significantly more active (swimming and crawling combined) than when they were injected with saline (paired *t*-test, *P *=* *0.0047, *t* = 14.56, degrees of freedom = 2, *n* = 3). Animals injected during the day did not show a significant change in behavior (*P *>* *0.05; *n* = 6 injected with both SCP_B_ and saline). When SCP_B_ was added to isolated brains that had not been producing the swim motor program for at least 5 min, the swimming rhythm was immediately initiated (*n* = 8; Fig. 5).

### Effects of SCP_B_ on the esophagus

**Fig. 6:**
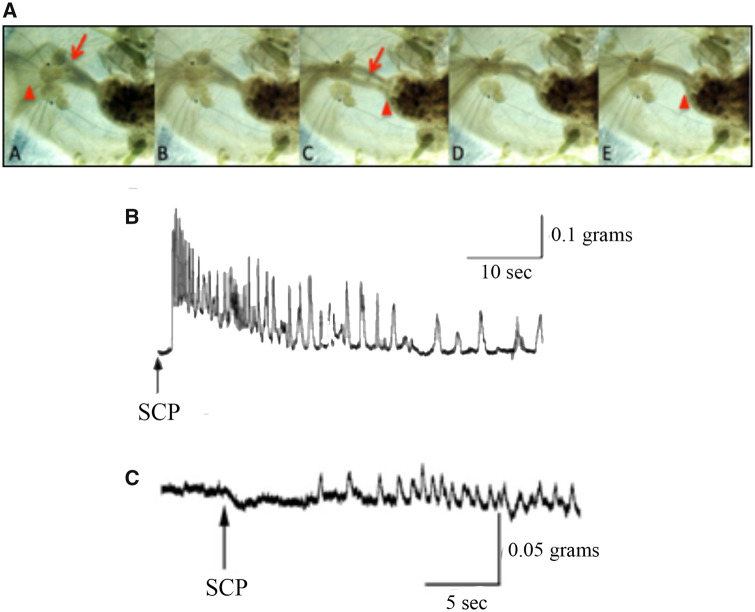
The influence of SCP_B_ on the *M. leonina* esophagus. (**A)** Sequential images from a semi-intact animal, showing the peristaltic contractions of the esophagus that are the basis of swallowing and are elicited by application of SCP_B_. (**B**) Recordings from a force transducer attached to the esophagus. The buccal ganglia innervate the esophagus and cause rhythmic contractions. When the buccal ganglia were still attached to the esophagus application of SCP_B_ elicited peristaltic contractions. (**C**) An example illustrating how SCP_B_ is also capable of inducing peristaltic contractions of an esophagus that did not have the buccal ganglia attached, indicating that it has direct effects on the esophageal musculature.

Application of 10^−5^ M SCP_B_ initiated peristaltic contractions of the esophagus with (*n* = 5), and without (*n* = 4), the buccal ganglia attached (Fig. 6).

## Discussion

The neuropeptide SCP_B_ was present throughout the CNS of *M. leonina*, and SCP_B_-like-immunoreactive processes were in a number of nerves that communicate with a variety of different tissues. Injection of SCP_B_ into intact animals enhanced crawling and swimming, and application of SCP_B_ excited isolated brains and the esophagus. Therefore, SCP_B_ appears to be a neuropeptide that causes a change in behavioral state and arousal, similar to octopamine and serotonin in crustaceans (Harris-Warrick and Marder 1991; Kravitz 2000) and serotonin in gastropods (Lloyd et al. 1984b; Katz et al. 2001; Jing et al. 2009).

SCP_B_-like immunoreactivity was present in almost two dozen neurons in the brain. These included a number of large neurons in the anterior region of the cerebral ganglia that are in a similar location as large serotonergic neurons (Newcomb et al. 2006). Serotonin and SCP_B_ colocalization has also been reported in insects (Homberg and Hildebrand 1989). It is also likely that one of the SCP_B_-like-immunoreactive neurons in the medial region of each cerebropleural ganglion is the C2 neuron, previously identified by Lillvis et al. (2012) as a homologue of the C2 neuron originally identified in *Tritonia diomedia* (Getting 1977; Taghert and Willows 1978). This neuron contains both SCP_B_ and FMRFamide (Lillvis et al. 2012) and, in some species that swim with dorsal–ventral flexions, is a member of the swim central pattern generator (Getting 1977; Taghert and Willows 1978; Jing and Gillette 1995). However, at this time, the role of the C2 neuron in *M. leonina* is not known.

There is a possibility that the monoclonal antibody to SCP_B_ that was used in this study might cross-react with the related neuropeptide, FMRFamide. There is evidence to suggest that this was the case in some prior studies with crustaceans and snails (Arbiser and Beltz 1991; Santama et al. 1994a). However, this monoclonal SCP_B_ antibody has been previously used to label SCP_B_ in nudibranchs (Masinovsky et al. 1988; Lillvis et al. 2012) and preadsorption controls with both SCP_B_ and FRMFamide suggest no such cross-reactivity occurs in nudibranchs, including *M. leonina* (Watson and Willows 1992). These monoclonal SCP_B_ antibodies may be more specific in these animals because they lack the extended forms of FRMFamide seen in other gastropods (Senatore et al. 2015).

*Melibe leonina* have a circadian rhythm of locomotion, including both crawling and swimming (Newcomb et al. 2014). The neurons that make up the central pattern generator for swimming have been identified and some of these, along with most of the swim motor neurons, are found in the pedal ganglia (Watson et al. 2002; Thompson and Watson 2005; Sakurai et al. 2014). The presence of many SCP_B_-containing varicosities and terminals in the pedal ganglia is consistent with the hypothesis that SCP_B_ plays an important role in modulating the expression of swimming behavior. This is further reinforced by the finding that application of SCP_B_ to isolated brains elicited expression of the swim motor program and injection of SCP_B_ into intact animals at night caused them to move further than control animals, with some of this elevated activity being due to more swimming.

In several gastropods, SCPs have been shown to enhance feeding motor programs as well as modulate the contractions of muscles associated with feeding, such as those that control movements of the buccal mass (Lloyd et al. 1984b; Murphy et al. 1985; Sossin et al. 1987; Willows et al. 1988; Miller et al. 1994). In many of these species, SCP_B_ is found in a number of buccal ganglion neurons, and in some cases they colocalize with other neurotransmitters, such as acetylcholine (Lloyd et al. 1988b; Whim and Lloyd 1989; Cropper et al. 1990), buccalin (Cropper et al. 1988; Vilim et al, 1996), glutamate (Klein et al. 2000), and myomodulin (Santama et al. 1994b). The buccal ganglion of *M. leonina* is much less complex than most other gastropod buccal ganglia because it swallows its food whole and does not grind it with the buccal mass. Therefore, it does not require the neural circuits that control movements of the radula and chewing musculature. The only SCP_B_-positive neuron in each of the *M. leonina* buccal ganglia is a large neuron that appears to be homologous to the B1 neuron in a number of other gastropod species (Watson and Willows 1992). This neuron is active during feeding and swallowing in *M. leonina*, as well as other species, and stimulation of this neuron has an excitatory effect in all species studied to date. In *M. leonina*, this neuron also projects to the stomach and innervates the esophagus, and therefore plays an important role in swallowing, and perhaps digestion as well (Watson and Willows 1992). The pharmacological data obtained in this study reinforce that conclusion. Application of SCP_B_ to isolated esophagus preparations elicited peristaltic contractions. Thus, SCP_B_ likely modulates other neurons in the buccal ganglia, as well as the esophageal musculature.

When SCP_B_ was injected into intact animals, it caused *M. leonina* to swim more often and crawl for a longer period of time at night, when they are typically more active (Newcomb et al. 2004; Newcomb et al. 2014), but not during the day. Furthermore, SCP_B_ elicited swim motor programs in isolated brain preparations, although spontaneous swim motor programs (Watson et al. 2001; Watson et al. 2002) cannot be ruled out as an alternative explanation for these results, given the experimental design. The likelihood that these swim motor patterns were a direct result of SCP_B_ is bolstered by a number of observations: (1) SCP_B_ was only added after preparations had been quiescent (i.e., no swim motor patterns) for extended periods of time (at least 5 min); (2) SCP_B_ always elicited a swim motor pattern and did so with a similar, relatively short latency; and (3) swimming was also increased when intact animals were injected with SCP_B_.

It is worth pointing out that, at night, *M. leonina* often “graze” on eelgrass and kelp, producing rhythmic feeding movements while they are also crawling (personal observation). Moreover, preliminary experiments indicate that injections of SCP_B_ into intact animals may also have a stimulatory effect on feeding (personal observation). Therefore, it is not surprising that SCP_B_ might modulate both types of behavior, in much the same manner as it modulates swimming, prey capture, and feeding in *Clione limacina* (Norekian and Satterlie 1994). We hypothesize that SCP_B_ changes the behavioral state in *M. leonina* so that it is more likely to swim, crawl, and feed at night. Therefore, our working hypothesis is that SCP_B_ is released more at night, than the day, and this release might be stimulated, in part, by the circadian clock.
